# A Study of Clinical Profile of Congenital Anomalies of Kidney and Urinary Tract (CAKUT) in a Tertiary Care Center

**DOI:** 10.7759/cureus.63766

**Published:** 2024-07-03

**Authors:** Anirudh Kommareddy, Keta Vagha, Jayant D Vagha, Chaitanya Kumar Javvaji, Amar Taksande, Revat J Meshram, Shailesh Wandile, Ajinkya Wazurkar

**Affiliations:** 1 Pediatrics, Jawaharlal Nehru Medical College, Datta Meghe Institute of Higher Education and Research, Wardha, IND

**Keywords:** vesicoureteral reflux, hypospadias, congenital anomalies, obstructive hydronephrosis, congenital heart disease

## Abstract

Background

Congenital anomalies of the kidney and urinary tract (CAKUT) encompass a diverse array of disorders arising from developmental irregularities in the renal parenchymal development, disrupted embryonic migration of the kidneys, and the urinary collecting system. This study aimed to investigate the clinical presentations, patterns of obstructive and non-obstructive CAKUT, and associated extrarenal manifestations in affected children.

Methods

This observational study was conducted in the Department of Pediatrics, Acharya Vinoba Bhave Rural Hospital, Wardha. Ethical clearance was obtained, and the study included 105 diagnosed CAKUT patients aged from birth to 18 years. Data collection spanned from June 2022 to May 2024. Clinical features, antenatal findings, associated anomalies, estimated glomerular filtration rate (eGFR), and serum creatinine levels were recorded. Descriptive and inferential statistical analyses were performed using Stata software.

Results

Among the 105 participants, 81 (77.14%) were males, with a male-to-female ratio of 3.37:1. The mean age was 42.49 months. Forty-two individuals (40%) were asymptomatic, while the most common symptomatic presentation was the ventral opening of the urethra (24.76%). Extrarenal malformations were present in 35 subjects (33.33%), with undescended testis (25.71%) and congenital heart disease (CHD) (20%) being the most common. The antenatal diagnosis was made in 63.8% of cases. Obstructive uropathy was present in 42.86% of subjects, with a significant association between antenatal diagnosis and bilateral hydronephrosis. Medical management was provided to 41.9% of subjects, while 58.1% underwent surgical interventions.

Conclusion

The study highlights the clinical variability and diverse presentations of CAKUT in children, with a substantial proportion being asymptomatic. Early detection through antenatal screening and prompt intervention can potentially prevent or delay the progression to ESRD. The findings underscore the importance of comprehensive evaluation and targeted management strategies to address both renal and extrarenal manifestations of CAKUT.

## Introduction

Approximately one in 500 live infants is affected with congenital anomalies of the kidney and urinary tract (CAKUT), and one in 2000 of such births results in neonatal death [1]. End-stage renal disease (ESRD) is primarily caused by CAKUT, which also accounts for 30% to 60% of pediatric ESRD patients. CAKUT is a significant cause of morbidity in children [2]. The name "CAKUT" describes a wide spectrum of diseases brought on by anomalies in the renal parenchymal formation, anomalies in the embryonic migration of the kidney or kidneys, and issues with the urine collection system. Ureterovesical junction obstruction (11%) and vesicoureteral reflux (VUR) (25%), among other lower urinary anomalies, account for almost 50% of people with the condition [3]. Renal anomalies account for 20-30% of all problems that can be detected and are frequently discovered during pregnancy [3].

The occurrence of syndromic varieties of CAKUT, familial clustering, and findings from animal models all indicate the essential role that genetic determinants play in the condition. A genetic basis is suggested by identifying over 50 single gene anomalies, representing 10-12% of isolated CAKUT cases [4]. CAKUT can present with a wide range of symptoms, from severe stages of renal failure to asymptomatic imaging abnormalities. Severe prenatal oligohydramnios may have a clinical history. Newborns with CAKUT may also have features similar to Potter's syndrome [5]. These features include unique facial features, including pseudoepicanthus, a receding chin, flat ears that are rotated posteriorly, a flat nose, reduced fetal movement, and anomalies of the musculoskeletal system such as clubfoot, hip dislocation, joint contractures, and hypoplasia of the lungs.

Consequently, our study aimed to investigate the variety of clinical presentations seen in kids with CAKUT. The main goal was to evaluate the clinical spectrum and presentation patterns in CAKUT. Other goals included investigating these patients' extrarenal symptoms and analyzing the clinical features of both obstructive and non-obstructive CAKUT.

## Materials and methods

Study design, setting, and study period

After receiving approval from the institutional ethics committee, the current observational study was conducted at the pediatric department of the Acharya Vinoba Bhave Rural Hospital, Wardha. The letter of approval that corresponds to it is DMIMS/(DU)/IEC/2022/1072. Data was gathered over two years, from June 2022 to May 2024.

Study participants

The study's inclusion criteria were all patients diagnosed with cases of CAKUT in the age group from birth to 18 years of age during the study period. Parents who do not give consent to the study are excluded from the study.

Ethics consideration and sample size

The DMIHER Institutional Review Board approved the study protocol with the number DMIMS(DU)/IEC/2022/1072. The sample size is determined using the following formula: n=(DEFF*Np(1-p)]/ [(d2/Z21-α/2*(N-1)+p*(1-p)), where N is the population size, p is the population, d denotes the exact percentage of 100 (absolute +/-%) for the confidence bounds, and DEFF is the design effect. In this study, 105 cases were considered.

Study procedure

**Figure 1:**
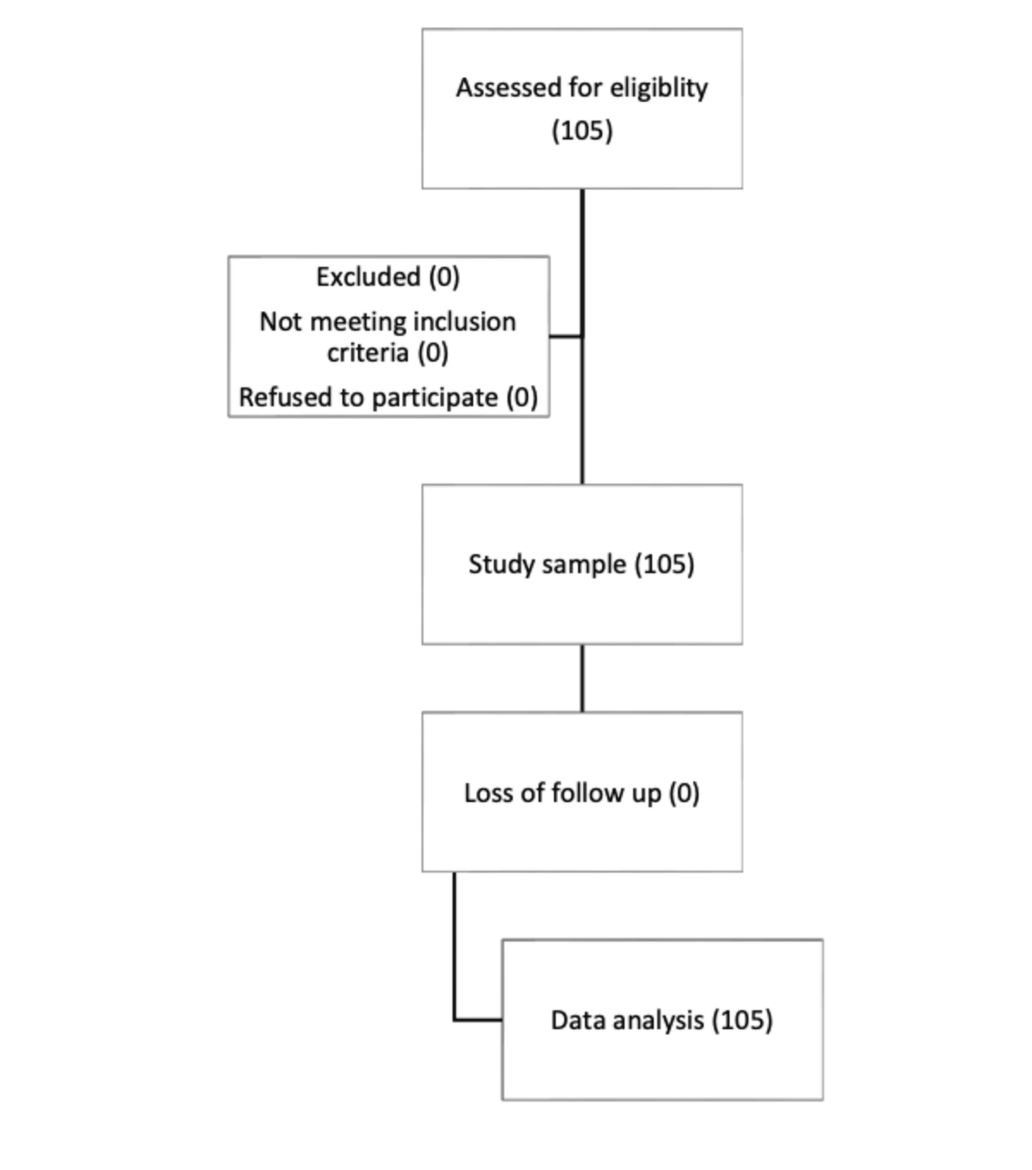
Flow chart of the study Figure by Dr. Anirudh Kommareddy

After ethical approval from the Committee, the purpose and objectives of the study were explained to parents or caregivers who fulfilled the section criteria. The informed consent form provided comprehensive information regarding the study, ensuring transparency and adherence to ethical guidelines. In order to ascertain the prevalence of CAKUT within our dependent community, all successive children who met the study's criteria and presented with the condition were included. All the sociodemographic details, serum creatinine levels, related anomalies, antenatal findings, clinical characteristics, and estimated glomerular filtration rate (eGFR) were noted. Weight and height measurements were also compared to WHO and the Indian Academy of Pediatrics charts. Blood pressure was checked using standard methods. Serum creatinine was measured in all patients. Using serum creatinine, eGFR is calculated using the formula 0.413×height÷serum creatinine. The study flow chart is in Figure 1.

Statistical analysis

Stata software was used for inferential and descriptive statistical analysis (Stata 10, StataCorp LLC, College Station, USA). While percentages and proportions were used to summarize qualitative data, mean, median, and standard deviation were used to analyze quantitative data. To compare means, the unpaired Student's t-test was utilized, with a significance level set at a p-value of less than 0.05.

## Results

**Figure 2:**
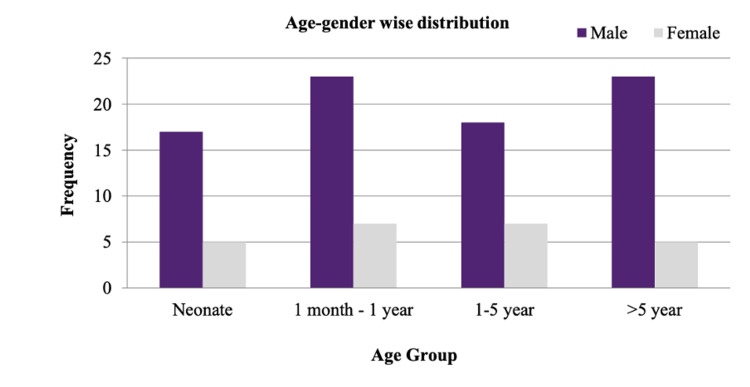
Bar diagram showing age- and gender-wise distribution of study sample

In our study, with a sample size of 105, the gender distribution was as follows: 81 males (77.14%) and 24 females (22.85%), resulting in a male-to-female ratio of 3.37:1, indicating a preponderance of males. The mean age of the 105 study sample was 42.49 months (standard deviation: 42.47 months), with the highest at 168 months and lowest at one day. In the study, 30 (28.57%) subjects were from the one-month to one-year age group, 28 (26.67%) subjects from the more than five years age group, 25 (23.81%) subjects from the one to five years age group, and 22 (20.95%) subjects from neonatal age group. Figure 2 shows the age- and gender-wise distribution of the study sample.

A significant portion, 42 individuals (40% of the total subjects), were asymptomatic, indicating that their condition did not manifest with any observable symptoms. Among those who did exhibit symptoms, the most common presentation was the ventral opening of the urethra, observed in 26 cases (24.76%). Fever was reported in 10 cases (9.52%), while abdominal distension was noted in nine cases (8.57%). Burning micturition (painful urination) and abdominal pain were each reported in five and four cases, respectively (4.76% and 3.81%). Dribbling of urine, hematuria (blood in urine), urinary tract infection, visible mass per abdomen, weak stream of urine, and whitish purulent micturition were less common, each observed in three or fewer cases (2.86% or less). These findings highlight the diverse clinical spectrum of CAKUT, with a substantial proportion of cases being asymptomatic but with a range of symptoms observed in those presenting with clinical manifestations (Table 1).

**Table 1:** Clinical presentation among study subjects

Clinical presentation	Number	Percentage
Asymptomatic	42	40.00%
Ventral opening of the urethra	26	24.76%
Fever	10	9.52%
Abdominal distention	9	8.57%
Burning micturition	5	4.76%
Pain abdomen	4	3.81%
Dribbling of urine	3	2.86%
Hematuria	2	1.90%
Whitish purulent maturation	1	0.95%
Week stream of urine	1	0.95%
Urinary tract infection	1	0.95%
Visible mass per abdomen	1	0.95%
Total	105	100.00%

**Figure 3:**
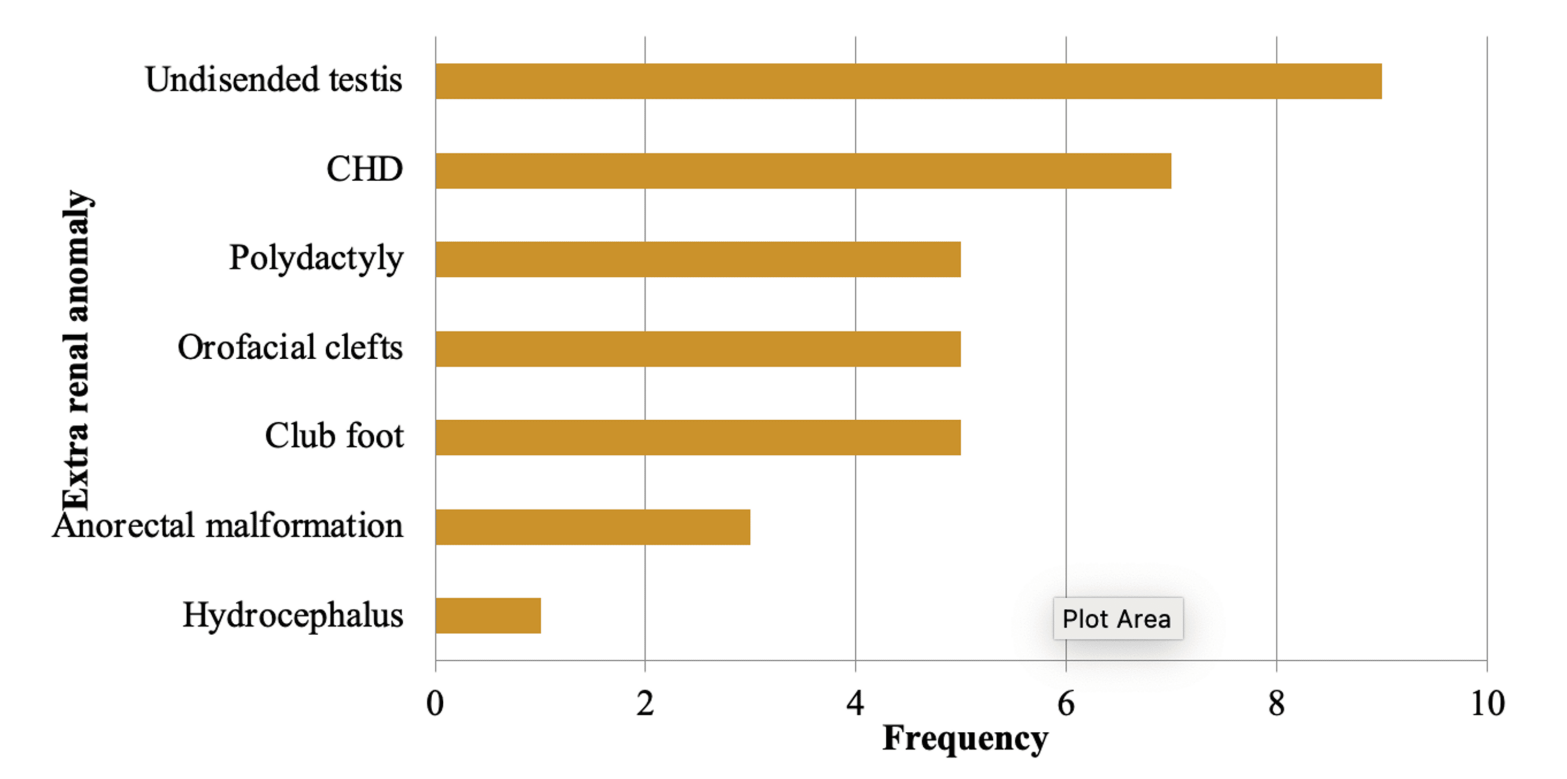
Bar diagram showing extrarenal anomalies among study subjects CHD, congenital heart disease Figure by Dr. Anirudh Kommareddy

Among the 105 subjects studied for CAKUT, a notable finding was that 70 individuals (66.7%) did not exhibit any extrarenal malformations. However, among the remaining 35 subjects who did have associated extrarenal malformations, the distribution was as follows: undescended testis was observed in nine cases (25.71% of subjects with extrarenal malformations), congenital heart disease (CHD) was present in seven cases (20%), club foot and orofacial clefts were each found in five cases (14.29% each), polydactyly was also observed in five cases (14.29%), anorectal malformation was noted in three cases (8.57%), and hydrocephalus was the least frequent, seen in one case (2.86%). This distribution showcases a range of extrarenal malformations associated with CAKUT, with undescended testis being the most common among this subgroup of subjects (Figure 3).

Table 2 shows extrarenal malformations among subjects.

**Table 2:** Extrarenal malformations among subjects CHD, congenital heart disease; ASD, atrial septal defect; PDA, patent ductus arteriosus; TOF, tetralogy of Fallot; VSD, ventricular septal defect

Extrarenal malformations	Number	Percentage
None	70	66.67%
Undiscended testis	9	8.57%
Club foot	5	4.76%
Orofacial clefts	5	4.76%
Polydactyly	5	4.76%
Anorectal malformation	3	2.86%
CHD- PDA	2	1.90%
CHD - ASD	1	0.95%
CHD - PDA	1	0.95%
CHD - TOF	1	0.95%
CHD - VSD	1	0.95%
CHD- VSD	1	0.95%
Hydrocephalus	1	0.95%
Grand total	105	100.00%

**Figure 4:**
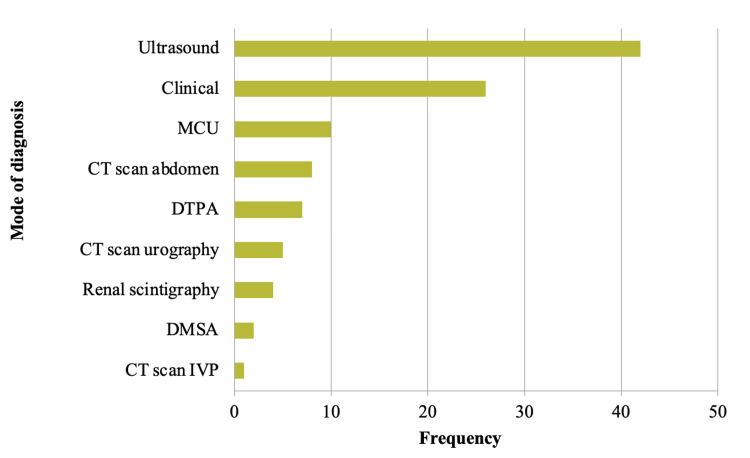
Bar diagram showing the mode of diagnosis among study subjects MCU, micturating cystourethrography; CT, computed tomography; DTPA, diethylene triamine pentaacetic acid; DMSA, dimercaptosuccinic acid, IVP, intravenous pyelogram Figure by Dr. Anirudh Kommareddy

The mode of diagnosis for CAKUT among the study subjects varied, reflecting a combination of clinical assessment and various imaging techniques. Among the 105 subjects, ultrasound emerged as the most commonly utilized diagnostic tool, with 42 cases (40% of the subjects) diagnosed through this method. Based on symptoms and physical examination, clinical diagnosis was also significant, accounting for 26 cases (24.76%). Micturating Cystourethrography (MCU) was utilized in 10 cases (9.52%), followed by a computed tomography (CT) scan abdomen in eight cases (7.62%) and diethylene triamine pentaacetic acid (DTPA) scans in seven cases (6.67%). CT scan urography and renal scintigraphy were employed in five and four cases, respectively (4.76% and 3.81%). CT scan intravenous pyelogram (IVP) and dimercaptosuccinic acid (DMSA) scans were relatively less frequently used, each noted in one to two cases. This comprehensive approach to diagnosis, combining clinical assessment with advanced imaging techniques like ultrasound and CT scans, ensures a thorough evaluation of CAKUT in these subjects. Figure 4 represents the mode of diagnosis among study subjects.

The diagnostic profile of the 105 study subjects with various CAKUT included a range of conditions. The ectopic kidney was identified in four subjects (3.81%), while two subjects (1.90%) had ectopic ureters with hydroureter. Other rare diagnoses included extrophy of the bladder (one subject, 0.95%), renal agenesis (one subject, 0.95%), and polycystic kidney disease (one subject, 0.95%). Hydronephrosis, characterized by the swelling of a kidney due to urine buildup, was prevalent among 49 subjects (46.67%), with two of them also exhibiting renal agenesis. Hydroureter, a condition involving dilation of the ureter, was noted in one subject (0.95%). In contrast, hydroureteronephrosis, a combination of ureter and kidney swelling, was found in 13 subjects (12.38%), including one with associated hypospadias and another with renal agenesis. Hypospadias, a condition where the urethral opening is on the underside of the penis, was diagnosed in 26 subjects (24.76%). Additionally, rare cases such as periureteric diverticuli (one subject, 0.95%), ureterocoele (one subject, 0.95%), and hydroureteronephrosis with renal agenesis (one subject, 0.95%) were also observed. These diverse diagnoses highlight the complexity and variability of CAKUT presentations among the study cohort. Table 3 shows the diagnosis among study subjects.

**Table 3:** Diagnosis among study subjects

Diagnosis	Frequency	Percentage
Hydronephrosis	49	46.67
Hypospadiasis	26	24.76
Hydroureteronephrosis	13	12.38
Ectopic kidney	4	3.81
Ectopic ureter with hydroureter	2	1.9
Hydronephrosis with renal agenesis	2	1.9
Extrophy of bladder	1	0.95
Hydroureter	1	0.95
Hydroureteronephrosis with hypospadias	1	0.95
Hydroureteronephrosis with renal agenesis	1	0.95
Paraureteric diverticuli	1	0.95
Polycystic kidney disease	1	0.95
Renal agenesis	1	0.95
Renal agenesis with hypospadias	1	0.95
Ureterocoel	1	0.95
Total	105	100

Out of the 105 subjects, 67 cases (63.8%) presented with an antenatal diagnosis of CAKUT, indicating that these anomalies were identified during prenatal screening or ultrasound examinations. On the other hand, 38 cases (36.19%) did not have an antenatal diagnosis, suggesting that their CAKUT condition was not detected until after birth or later in life. All the subjects had access to proper antenatal care and screening facilities. Among the study subjects, obstructive uropathy was examined, with 45 cases (42.86%) presenting with this condition and 60 cases (57.14%) showing an absence of obstructive uropathy.

The analysis showed no significant association between gender and obstructive uropathy (p=0.737). Both males and females were affected, with 34 males and 11 females presenting with obstructive uropathy. Regarding antenatal diagnosis, seven subjects who were not diagnosed antenatally were found to have obstructive uropathy. This also shows a significant association (p=0.000) as patients with antenatal diagnosis were more likely to have obstructive uropathy compared to those diagnosed postnatally. The analysis also showed a significant association between bilateral hydronephrosis and obstructive uropathy (p=0.001). Patients with bilateral hydronephrosis were more likely to have obstructive uropathy compared to those without bilateral hydronephrosis. Specifically, 15 cases (75%) with bilateral hydronephrosis had obstructive uropathy, while only 30 cases (35%) without bilateral hydronephrosis had this condition.

The mean serum creatinine level for individuals with obstructive uropathy was determined to be 0.8244 mg/dL, with a standard deviation of 1.58245 mg/dL. In the absence of obstructive uropathy, the mean serum creatinine level was slightly higher at 0.9983 mg/dL with a standard deviation of 1.40983 mg/dL. The F value for serum creatinine was 0.009, and the associated p-value was 0.554, indicating no statistically significant difference in serum creatinine levels between subjects with and without obstructive uropathy. Subjects with obstructive uropathy had a mean eGFR of 76.5969 mL/min/1.72m^2^ with a standard deviation of 40.34080 mL/min/1.72m^2^. In contrast, subjects without obstructive uropathy had a mean eGFR of 65.4797 mL/min/1.72m^2^ with a standard deviation of 39.56072 mL/min/1.72m^2^. The F-value for eGFR was 0.329, and the associated p-value was 0.161, indicating no statistically significant difference in eGFR between subjects with and without obstructive uropathy.

Among the 105 study subjects with CAKUT, the management approach encompassed both medical and surgical interventions. Forty-four subjects (41.90%) received medical management, likely including conservative treatments such as medication, lifestyle adjustments, and regular monitoring. Surgical management was undertaken for 61 subjects (58.10%), with procedures tailored to specific conditions. Twenty-six subjects (24.76%) underwent hypospadias repair to correct the positioning of the urethral opening. Anderson hynes pyeloplasty, performed in 22 subjects (20.95%), addressed blockages between the kidney and ureter. Additional surgical procedures included cystoscopy (one subject, 0.95%), Double-J (DJ) stenting (two subjects, 1.90%), nephrectomy (two subjects, 1.90%), ureteric reimplantation (two subjects, 1.90%), pelvic osteotomy with primary repair of bladder exstrophy (one subject, 0.95%), and PUV fulguration (five subjects, 4.76%). These interventions aimed to correct anatomical abnormalities, improve urinary function, and alleviate associated complications.

## Discussion

Demographics

In the study sample, males were more commonly affected by CAKUT than females, with a ratio of 3.37:1. This finding aligns with previous research. Li et al. found that males had a higher risk of CAKUT than females, with a sex ratio of 1.44:1 [6]. Similarly, Tain et al. observed that males had a 1.83-fold more significant risk of CAKUT than females [7]. This aligns with existing literature that suggests certain congenital kidney and urinary tract anomalies are more common in males. The gender disparity underscores the need for targeted screening and early intervention strategies, particularly in male neonates and infants. The age distribution was relatively even across different age groups, with the highest representation among infants (28.57%). The high prevalence in infants may reflect the importance of neonatal screening programs and good clinical examination. This demographic trend emphasizes the importance of early diagnostic evaluations and continuous monitoring from birth.

Clinical presentation 

Though most patients were asymptomatic at the time of diagnosis (40%, n=42), thorough clinical examination revealed that the ventral opening of the urethra was the most common observed anomaly (n=26), mainly hypospadias, indicating the presence of underlying CAKUT. Fever was the most common symptom prompting further investigation. Burning micturition, hematuria, and urinary stream abnormalities were less common. Hydronephrosis (n=49, 46.67%), hypospadiasis (n=26, 24.76%), and hydroureteronephrosis (n=13, 12.38%) were the most prevalent anomalies. Less common anomalies included ectopic kidney, bladder exstrophy, polycystic kidney disease, and renal agenesis. Chaara et al. found that multicystic dysplastic kidney (MCDK) was the most common fetal abnormality, followed by PUV, renal agenesis, and others [8]. Chougule et al. observed that CAKUT were more prevalent in males, with hydronephrosis being the most frequent CAKUT detected prenatally [9]. They also noted that a significant percentage of mild hydronephrosis cases resolved postnatally within a few days, and a notable portion resolved within six months of age.

Extrarenal anomalies 

We also analyzed extrarenal anomalies in our cohort of 105 babies. Among these infants, 70 (66.67%) were found to have no extrarenal malformations. The most prevalent malformation was undescended testis, observed in nine cases (8.57%). This was followed by club foot, orofacial clefts, and polydactyly, each noted in five cases (4.76%). Anorectal malformation was identified in three cases (2.86%). Various CHDs, including patent ductus arteriosus, atrial septal defect, tetralogy of Fallot, and ventricular septal defect, were each reported in one case (0.95%). Additionally, hydrocephalus was observed in one case (0.95%).

According to Li et al., limb abnormalities were the second most prevalent anomaly linked to CAKUT after congenital cardiac problems [6]. According to other research, the genitourinary tract, central nervous system, musculoskeletal system, digestive system, cardiovascular system, and chromosomal abnormalities are the main systems affected by non-urinary defects [10]. An estimated 0.3 to 2.1 out of every 10000 live newborns are thought to have the VACTERL association, which is characterized by a combination of vertebral anomalies, anal atresia, cardiac malformations, tracheoesophageal fistula, renal anomalies, and limb abnormalities [11].

Although our study did not have vertebral anomalies, a more extensive study group might reveal more associated extrarenal anomalies. Alp et al. evaluated 806 children with CAKUT, performing transthoracic echocardiography on 135 patients, and found CHD in 91 cases (11.2%) [12]. The most common CHD was an atrial septal defect, found in 80.2% of patients, whereas our study had patent ductus arteriosus as the most common anomaly. The majority of participants did not have extrarenal malformations (66.67%). However, undescended testis, clubfoot, oro-facial clefts, and polydactyly were the most prevalent among those with additional anomalies. This suggests a pattern where certain extrarenal malformations may co-occur with congenital urological anomalies, necessitating a comprehensive clinical assessment for affected individuals to address all potential health concerns.

Investigations and diagnosis 

Our study found that 67 cases (63.8%) presented with an antenatal diagnosis of CAKUT, indicating that these anomalies were identified during prenatal screening or ultrasound examinations. On the other hand, 38 cases (36.19%) did not have an antenatal diagnosis, suggesting that their CAKUT condition was not detected until after birth or later in life. Prenatal ultrasounds successfully diagnosed CAKUT in 60-85% of babies, according to Murugapopathy et al. [13]. This accuracy was higher when imaging was done in the third trimester. Prenatal ultrasonography was also proven to be beneficial for post-diagnosis and postnatal surgical treatment by Bhide et al. [14]. While the detection rates for VUR, duplex renal system, and periurethral junction obstruction were 82.8%, 67%, and 26.1% respectively, the detection rate for multicystic renal dysplasia was 100%. They discovered that the kind of renal tract pathology affected the prevalence of aberrant results on prenatal ultrasounds.

Richter-Rodier et al. compared ultrasound performed antenatally and postnatally and found that CAKUT cases were detected in 3.7% of kidneys during ultrasound exams, 18.2% prenatally, 65.2% in three to seven days postnatally, and 17% during a six-month follow-up [15]. Their study also highlighted a male predominance, with hydronephrosis being the most common obstructive nephropathy (83.3%). Postnatal ultrasound screening showed higher diagnostic sensitivity (79.6%) compared to prenatal (18.2%), with specificity consistently above 99%. Ultrasound emerged as the most frequently used diagnostic tool (40%), followed by clinical examination (24.76%). The reliance on ultrasound is consistent with its non-invasive nature, accessibility, and effectiveness in identifying urological abnormalities. These diagnostic preferences underline the need for maintaining and improving access to high-quality ultrasound equipment and trained personnel in clinical settings.

The gold standard dynamic test for the diagnosis of fetal uropathy diagnosed antenatally or for the workup of VUR is the voiding cystourethrogram. A DMSA scan is therefore recommended in situations of scarring, pelvic or thoracic kidney cases, crossing fused ectopia, duplex kidney, MCDK, horseshoe kidney, and any information regarding the location of the working parenchyma that justifies static nuclear imaging. For blocked systems, including pelvic ureteric junction blockage and obstructive megaureters, the dynamic isotope study, DTPA or 99mTc, ethylene dicysteine scan, should be conducted. This paper covers the kidney's vascular, intrarenal, and excretory phases in detail [16].

Crucially, CT and magnetic resonance imaging aid in the early detection of problems such as renal calculi, infections, and cancers in addition to confirming abnormalities found by ultrasonography, identifying complicated anomalies, and illustrating the collecting system and vascular anatomy [17].

Obstructive versus non-obstructive conditions 

Among the 105 study subjects, obstructive uropathy was present in 45 cases (42.86%), while it was absent in 60 cases (57.14%). Further analysis revealed that males presented more often with obstructive uropathy than females, but this gender comparison was not statistically significant. However, the age comparison showed that diagnoses made below one year of age and between one and five years were statistically significant. Antenatal diagnosis significantly correlated with obstructive uropathy, with patients diagnosed prenatally more likely to have this condition compared to those diagnosed postnatally (p=0.000). Additionally, a significant association existed between bilateral hydronephrosis and obstructive uropathy (p=0.001), with 75% of cases with bilateral hydronephrosis presenting with obstructive uropathy compared to 35% without bilateral hydronephrosis.

Kumar et al. compared obstructive uropathy and non-obstructive uropathy cases, revealing statistically significant findings [2]. The antenatal diagnosis was significantly more prevalent among cases with obstructive uropathy (89.5% vs. 76.9%, p=0.01). Male sex showed a significant association with these conditions (65.2% vs. 63.3%, p=0.02), unlike our study. Urinary tract infections (UTIs) were notably more common in affected individuals (5.8% vs. 12.6%, p<0.01). Moreover, individuals with renal and urinary tract abnormalities exhibited significantly lower eGFR (median eGFR: 60 vs. 62.8, p<0.01) [2]. Serum creatinine and eGFR, however, did not change substantially between the two groups in our investigation (p>0.05). Interestingly, those with these abnormalities had earlier diagnosis ages (median age: seven months vs. 10 months, p=0.01). The results above emphasize the significance of timely identification via prenatal screening, especially for males, and draw attention to the heightened likelihood of urinary tract infections and impaired kidney function linked to these ailments. As a secondary inflammatory process, interstitial inflammation, cortical cysts, glomerular hyalinization, and reduced glomerular number inside the kidney are all possible outcomes of obstructive uropathy [18].

Nevertheless, despite having normal or almost normal eGFR and serum creatinine, Biswas et al. showed that a significant percentage of children with PUV, bilateral reflux, or reflux in one kidney had poor development and hypertension [19]. Non-obstructive uropathy included diseases including renal agenesis and multicystic kidney disease, which can result in kidney loss and decreased eGFR. Therefore, growth parameters should be included in any future studies to provide a more comprehensive understanding of the impact of CAKUT on pediatric patients. These results emphasize the critical role of thorough clinical evaluation and the use of advanced imaging techniques in the early detection and management of obstructive uropathy and the necessity of ongoing monitoring to prevent long-term complications.

Obstructive uropathy was significantly associated with age group and antenatal diagnosis, indicating that younger age groups and those diagnosed antenatally are more likely to have obstructive conditions. Additionally, bilateral hydronephrosis was significantly more common in obstructive cases, emphasizing the need for thorough evaluation in these patients. Renal function is evaluated using eGFR and serum creatinine. The renal function test included a measurement of serum creatinine. The formula to determine eGFR is 0.413×height÷serum creatinine. It was discovered that non-obstructive uropathy patients had a slightly increased mean serum creatinine level. On the other hand, non-obstructive uropathy was shown to have a somewhat lower mean eGFR. Interestingly, the difference in serum creatinine and eGFR levels between those with and without obstructive uropathy is not statistically significant. This suggests that renal function may not differ significantly at diagnosis but still requires close monitoring.

Management 

Medical management was the most common treatment approach (41.9%), reflecting its primary role in managing less severe cases and symptomatic relief. Surgical interventions, including hypospadias repair, Anderson-Hynes pyeloplasty, and PUV fulguration, were also critical in managing more complex cases. This study provides valuable insights into the demographics, clinical presentations, diagnostic methods, and treatment approaches for congenital urological anomalies. The findings emphasize the importance of early diagnosis, mainly through antenatal screening, and highlight the need for a multidisciplinary approach to manage these conditions effectively.

## Conclusions

The study highlights the wide range of clinical manifestations and clinical variability of children's CAKUT defects. A significant fraction of those afflicted have no symptoms at all, which highlights the role prenatal screening plays in early identification. The necessity for a comprehensive approach to patient evaluation and care is highlighted by the fact that extrarenal symptoms are present in a significant proportion of cases. The results show that the illness can be effectively managed, and the advancement of ESRD can be prevented or delayed with timely and suitable medicinal or surgical therapies. This study emphasizes how crucial it is to provide early diagnosis and individualized treatment programs in order to enhance the quality of life and long-term results for kids with CAKUT.
